# Regenerative Endodontic Procedures of Immature Permanent Premolars with Periapical Lesions: A Report of Two Cases Using Two Different Materials, 18-Month Follow-Up

**DOI:** 10.1155/2023/5577474

**Published:** 2023-11-20

**Authors:** Hong Van Le, Tuan Anh Nguyen, Thai Son Vu

**Affiliations:** ^1^Department of High Technology Dental Treatment, National Hospital of Odonto-Stomatology, Hanoi, Vietnam; ^2^Department of Endodontics, National Hospital of Odonto-Stomatology, Hanoi, Vietnam; ^3^Endodontic Department, Dental Faculty, Hanoi University of Business and Technology, Hanoi, Vietnam

## Abstract

Regenerative endodontic procedure is an emerging alternative to traditional therapies for immature teeth with necrotic pulp with or without periapical lesions. This innovative approach, also known as revitalization procedures, is aimed at enhancing canal wall thickness, stimulating root lengthening, and promoting apical closure. The regenerative endodontic procedures involve minimally invasive cleaning to preserve stem cells, stimulation of bleeding and clot formation within the canal, and the use of biomaterials to stimulate differentiation. This method is the first choice in biologically based treatments for immature permanent teeth. We present two successful clinical cases in which regenerative endodontic procedures were performed on permanent premolars with necrotic pulp with symptomatic apical periodontitis and chronic apical abscess due to dens evaginatus. The same procedure was employed for both cases, utilizing two differential materials: ProRoot MTA (Dentsply Tulsa Dental Specialities, USA) and Biodentine™ (Septodont, Saint-Maur-des-Fossés, France). Both cases exhibited positive clinical and radiographic outcomes after an 18-month follow-up period including periapical healing, increased dentin thickness, root lengthening, and apical closure.

## 1. Introduction

Treatment of immature permanent teeth with necrotic pulp presents a significant clinical challenge in endodontics. Traditional treatment methods such as multiple-session apexification using calcium hydroxide and one-step apexification with biomaterials (such as MTA) only create a mineralization barrier at the apex but do not stimulate root growth, leading to the risk of fragile roots [1].

Recently, a biological treatment approach has been focused on creating an environment that stimulates root maturation. This involves a minimally invasive cleaning of the root canal and the use of an intracanal medication to address periapical lesions. The root canal is reperfused by inducing bleeding from the apex, followed by the application of a biomaterial to the clot. This method was initially named revitalization and then regenerative endodontics. Regenerative endodontics is a biological concept that relies on three key elements, stem cells, scaffold, and growth factors, to regenerate the pulp–dentin complex, thereby promoting root growth, apical closure, dentin formation, and healing of periapical lesions [2–6]. Despite numerous studies demonstrating successful clinical and radiographic outcomes, the efficacy of the regenerative endodontic procedures (REPs) in case of apical periodontitis still lacks robust scientific evidence [7]. The viability of Hertwig's epithelial root sheath (HERS) and the apical papilla plays a crucial role in the success of REPs, as it regulates stem cell activity through complex epithelial–mesenchymal interactions [8]. Several studies propose that the apical papilla and HERS/ERM persist and undergo differentiation to successfully develop a tooth within the REPs even after endodontic infection [9]. The regenerative endodontic protocol emphasizes minimally invasive cleaning of the root canal system, scaffolding by inducing blood flow from periapical tissue into the root canal, and stimulation of clot formation containing activated stem cells in the root canal, followed by crown sealing to prevent reinfection [10].

This article reports two clinical cases of mandibular premolars with pulp necrosis due to dens evaginatus and periapical lesions. These cases were treated by regenerative endodontic methods with minimally invasive cleaning, using two different materials ProRoot MTA and Biodentine™, followed up by 18 months.

## 2. Case Presentation

### 2.1. Case 1

**Figure 1:**
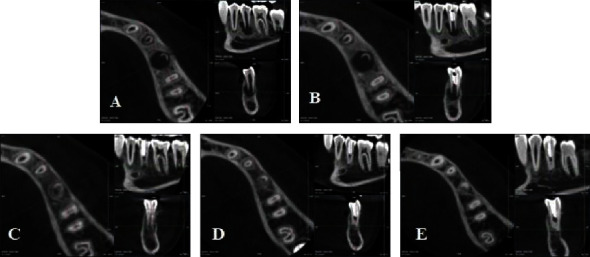
(A) Before treatment. (B) Immediately after tissue regeneration. (C) 6-month follow-up. (D) 12-month follow-up. (E) 18-month follow-up.

**Figure 2:**
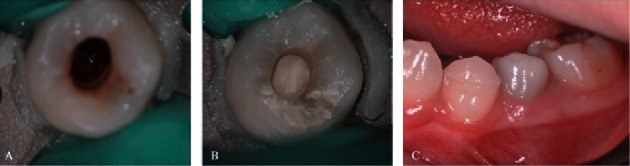
(A) Blood clot inside the canal. (B) MTA placement. (C) Discoloration of tooth #35 observed after 12 months.

A 12-year-old male presented with a dental fistula associated with the second lower left premolar suffered 6 months ago. Clinical examination revealed the presence of dens evaginatus on tooth #35 which was treated through grinding. The tooth exhibited painless to percussion and palpation; vitality test yielded a negative result. Cone-beam computed tomography (CBCT) images showed an open apex (Cvek stage II) and periapical lesions with the resorption through the buccal cortical bone (Figure 1(A)). Based on these findings, tooth #35 was diagnosed with pulpal necrosis and chronic apical abscess which has yet to achieve complete apex closure due to the dens evaginatus. The recommended approach, as per the European Society of Endodontology (ESE) guidelines, indicated REPs using ProRoot MTA (Dentsply Tulsa Dental Specialities, USA) over three appointments: two for endodontic regeneration and one for crown restoration [11]. During the first intervention, following the placement of the rubber dam, tooth #35 was accessed, the working length was determined by Intra-Oral Periapical Radiography with K file No. 10 (Dentsply Maillefer, Ballaigues, Switzerland), and the root canal was irrigated with 1.5% NaOCl and ultrasonic activation. Calcium hydroxide (UltraCal, Ultradent Inc., Utah, USA) was applied for 3 weeks, and temporary filling was installed with Fuji IX (GC, Japan). Upon the patient's return after 3 weeks, there were no evident symptoms or fistula. Local anesthesia was performed with Scandonest 3% (Septodont, Saint-Maur-des-Fossés, France), and a rubber dam was placed. The temporary filling on tooth #35 was removed, and the root canal was cleaned with 20 ml EDTA 17% (Coltene, USA). A K file No. 25 (Dentsply Maillefer, Ballaigues, Switzerland) with a 3 mm bended tip was employed, and a 10-minute clot formation period was observed under the microscope. CollaCote (Coltene, Ohio, USA) was placed over the clot (Figure 2(A)), with ProRoot MTA subsequently placed up to 2 mm from the cemento-enamel junction (Figures 1(B) and 2(B)). After 48-hour intervals, crown restoration was carried out using Fuji IX and composite (Tokuyama Dental Corp., Tokyo, Japan). At the 6-month, 12-month, and 18-month follow-up examinations, the patient underwent clinical reevaluation, dental pulp testing, and CBCT imaging (Figures 1(C)–1(E)). The follow-up examination revealed an absence of clinical symptoms and no sensitivity to percussion or palpation on tooth #35. Positive results were obtained from both electric and cold tests after 12 months, with stability observed after 18 months. However, there was a noticeable darkening of the crown after 12 months (Figure 2(C)). The CBCT image taken after 6 months showcased complete healing of the periapical lesion, presence of radiopaque tissue resembling dentin within the canal, elongation of the root, and advancement in apex closure from Cvek stage II to stage III. After 12 months, significant increase in root length and thickness of dentinal wall as well as complete apex closure (Cvek stage V) were observed on CBCT. These results remained stable at 18-month mark.

### 2.2. Case 2

**Figure 3:**
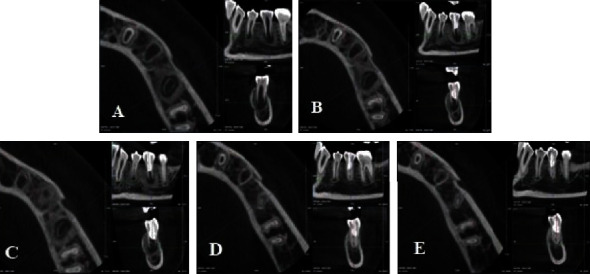
(A) Before treatment. (B) Immediately after tissue regeneration. (C) 6-month follow-up. (D) 12-month follow-up. (E) 18-month follow-up.

**Figure 4:**
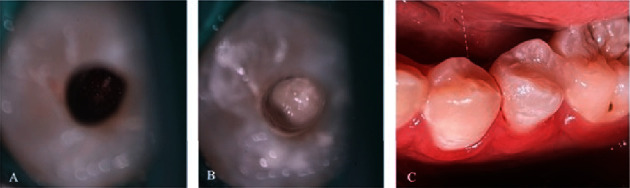
(A) Blood clot inside the canal. (B) Biodentine™ placement. (C) Clinical image after 18 months.

A 12-year-old female presented with acute pain in the left lower jaw and swelling in the vestibule corresponding to the premolars. The percussion and palpation tests yielded positive results. Tooth #35 exhibited worn dens evaginatus on the occlusal surface, with negative responses from the electric and cold pulp tests. CBCT imaging revealed lesions around the apex of tooth #35, with the apex remaining unclosed (Cvek stage II) (Figure 3(A)). The patient was diagnosed with pulpal necrosis and symptomatic apical periodontitis on tooth #35, which had not yet achieved complete apex closure due to the presence of dens evaginatus. REPs using Biodentine™ (Septodont, Saint-Maur-des-Fossés, France) were recommended following the two-appointment process outlined by the ESE protocol [11]. The first appointment followed the same procedure as described in case 1. During the second appointment (Figure 4(A)), the crown restoration was immediately performed once the Biodentine™ had been set, using GIC and composite materials (Figures 3(B) and 4(B)). The patient underwent clinical reexamination, dental pulp testing, and CBCT imaging at 6 months (Figure 3(C)), 12 months (Figure 3(D)), and 18 months (Figure 3(E)) posttreatment. The follow-up examination revealed the absence of clinical signs, with negative results from percussion and palpation test on tooth #35. Positive responses were obtained from both electric and cold pulp tests after 12 months, and these results remained stable at the 18-month recall. The crown did not exhibit any color changes (Figure 4(C)). The CBCT image taken at 6 months showed a reduction in the size of the periapical lesion. There was a density of radiopaque tissue within the canal and resembling dentin, and the roots exhibited elongation. At the 12-month CBCT, significant elongation of the roots, thickening of root dentin, and complete apex closure (Cvek stage V) were observed. These outcomes remained stable at the 18-month follow-up.

## 3. Discussion

This study shows two cases of symptomatic apical periodontitis and chronic apical abscess on tooth #35 having necrotic pulp with incomplete apical closure, classified as Cvek stage II. Both patients were treated by regenerative endodontics following the ESE protocol, using two different biomaterials, which resulted in similar clinical and radiological outcomes after 18 months. After the first intervention, all symptoms of apical periodontitis were resolved. The 6-month follow-up CBCT images showed that the periapical lesion was completely healed. According to the meta-analysis results of Tong et al. and He et al., more than 90% of the periapical lesions have healing signs after REPs [12, 13]. On the contrary, Meschi et al. in a systematic review concluded the lack of scientific evidence regarding the role of REPs in cases of periapical inflammation [7]. Therefore, it is important to acknowledge that further scientific evidence is necessary to establish the role of REPs in such cases.

In both cases, REPs resulted in root elongation, thickening of the root dentin wall, complete apex closure after 12 months, and positive response from cold and electric pulp tests. These outcomes can be attributed to the survival of Herwig's epithelial root sheath. Stem cells from apical papilla (SCAPs) within the blood clot are differentiated into odontoblast-like cells under the influence of biomaterials (ProRoot MTA in case 1 and Biodentine™ in case 2) [14]. These cells contribute to root formation and development after resolution of the periapical lesion [9].

A study by Sonoyama et al. in 2006 revealed that the dental papilla serves as a reservoir of SCAPs for the development of immature teeth [15]. These cells are capable of surviving and developing in hypoxic and hypoperfused environments and are nourished through passive permeation from surrounding tissues. The presence of a well-established blood supply system in periapical granulomas facilitates the growth of SCAPs. This study also shows that Herwig's epithelial cells play a crucial role in controlling stem cell differentiation during cementum formation and the transition of periodontal ligament into cementoblast, as demonstrated in in vitro studies [16]. Root growth and development following tissue regeneration are not solely dependent on HERS cells but also on the survival of SCAPs adjacent to the root after infection removal. However, according to the histological studies on animals and humans, it has been observed that stem cells differentiate into periodontal tissue rather than pulp tissue due to the presence of a cellular cementum on the dentin wall, bone, and periodontal ligament [9, 17, 18]. In our cases, both chronic and acute periodontitis lesions exhibited completed apex closure; after 12-month tissue regeneration, the apex was completely closed, increased root length, and thickened root dentin after 12-month tissue regeneration.

The use of Biodentine™ or ProRoot MTA did not significantly impact treatment outcomes in these two cases. Studies on dentinogenesis and pulp tissue survival and endodontic regeneration have shown that both materials, ProRoot MTA and Biodentine™, are effective in dentinogenesis and root development. However, Biodentine™ exhibits superior color stability compared to MTA [19, 20].

The Cvek classification accurately evaluates the root growth and apical closure on radiographs. Cvek stage V indicates complete root development. According to Alghamdi and Alsulaimani, only 45% of successful REP cases showed root development maturation with completed apex closure (Cvek stage V) [21]. In our report, both cases demonstrate progression from Cvek stage II to stage V after treatment.

Our endodontic regeneration treatment was performed according to the guidelines of ESE, in which the minimally invasive shaping and cleaning of the canal were performed with 1.5% NaOCl. Studies have shown that the concentration of NaOCl solution affects the survival and differentiation of SCAPs cultured in hyaluronic acid after 7 days, with 1.5% NaOCl having minimal negative effects compared to 3% or 6% NaOCl [22, 23]. EDTA 17% has demonstrated positive effects on the survival and differentiation of SCAPs. The use of EDTA solution in the final step further reduced the harmful effects of NaOCl in the previous steps [24]. In addition to the effects of irrigants, many authors are interested in the effects of intracanal medications. In the literature, triple-antibiotic combination (ciprofloxacin, metronidazole, and minocycline), dual-antibiotic combination (ciprofloxacin and metronidazole), and calcium hydroxide have been used in studies of endodontic regeneration. These studies have found that there are adverse effects on stem cell survival and differentiation of antibiotics while calcium hydroxide has a stimulating effect on stem cell growth [25].

The time of final restoration was different in two cases. In case 2, it was completed right after Biodentine™ completely set with GIC and composite. As per the research conducted by Palma et al., it has been found that both Biodentine™ and TotalFill BC Putty are highly suitable for promptly sealing the tooth crown following pulp capping. These materials demonstrate superior adhesive strength in the restoration of the tooth structure compared to other calcium silicate cements [26].

Successful REPs, as defined by the ESE guidelines, include (1) the absence of inflammatory symptoms, (2) radiographic evidence of bone healing, (3) increased root length and thickness, (4) absence of external root resorption, (5) positive response to the dental pulp test, (6) signs of new periodontal ligament formation, and (7) unchanged crown color [11]. In the American Association of Endodontists (AAE) guidelines, the success of REPs is measured by three levels of goal: (1) primary goal: elimination of inflammatory symptoms and evidence of bone healing; (2) the second goal: increase in root length and wall thickness; and (3) the tertiary goal: positive response to dental pulp testing [27]. In the published studies, the rate of positive response to electric pulp tests is only 50%-60%; some studies did not record a vital response of the pulp after REPs [28, 29]. However, in our two 18-month follow-up clinical cases, almost all AAE and ESE criteria groups were met except for tooth discoloration in case 1. According to Palma et al.'s study, the interaction between blood clot and biomaterial is a crucial factor in tooth discoloration. MTA causes more discoloration than Biodentine™; therefore, they suggested that the selection of biomaterial in REPs should consider this discoloration characteristic [19, 30].

## 4. Conclusion

The report of these two cases demonstrates complete apical healing and continued root formation in immature permanent necrotic teeth with apical periodontitis managed by REPs. However, an unfavorable outcome related to tooth discoloration was observed. The successful treatment of immature permanent teeth with necrotic pulp and apical periodontitis using REPs highlights the potential of this conservative approach. Nevertheless, further research is necessary to ensure consistent outcomes, enhance clinical protocols, and contribute to the broader adoption and effectiveness of REPs in dental practice.

## Data Availability

The data used to support the findings of this study are available from the corresponding author upon request.
